# 2348. Identifying Barriers Against COVID-19 Vaccination: A Prospective Study

**DOI:** 10.1093/ofid/ofad500.1970

**Published:** 2023-11-27

**Authors:** Gabriela S Generette, William Graft, Kayeromi Gomez, Basil Muhan, Jeana Kim, Kovas Polikaitis, Megan Price, Samantha Aguilar, Moamen Al Zoubi

**Affiliations:** Mercyhealth Internal Medicine Residency Program, Machesney Park, Illinois; University of Illinois Medicine, Rockford, Illinois; University of Illinois Medicine, Rockford, Illinois; University of Illinois Medicine, Rockford, Illinois; University of Illinois Medicine, Rockford, Illinois; University of Illinois Medicine, Rockford, Illinois; University of Illinois Medicine, Rockford, Illinois; University of Illinois Medicine, Rockford, Illinois; Infectious Disease Department/ Mercyhealth Internal Medicine Residency Program, Rockford, Illinois

## Abstract

**Background:**

As of April 26th 2023, there have been 104,445,294 reported cases of COVID-19 in the United States. The percentage of fully vaccinated people in the U.S is 69.4%, despite wide availability of COVID-19 vaccines. In Winnebago County, IL only 60.2% of the population is fully vaccinated. The medical community’s approach towards increasing the rate of vaccination has necessitated understanding which factors persist that prevent a greater willingness to vaccinate.

**Figure:**
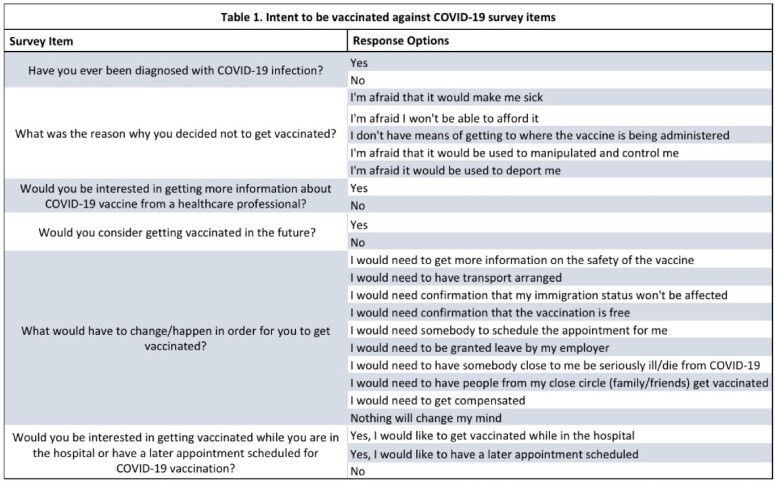

Intent to be vaccinated against COVID-19 survey items

**Figure:**
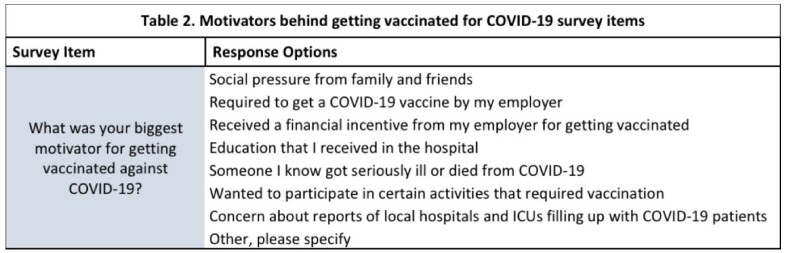

Motivators behind getting vaccinated for COVID-19 survey items

**Methods:**

Between June 2022 to March 2023, a daily report from the Electronic Medical Record (EMR) showing COVID 19 vaccination status was generated. The report was filtered to identify patients who did not have COVID-19 vaccine doses logged in the EMR. A medical student thenadministered the study survey to each of these patients. Patients who chose not to participate in the study were excluded. Patients also had an opportunity to discuss their concerns about COVID-19 infection and/or vaccination.

**Results:**

A total of 120 patients were consented for this study, with 73.33% (n=88) self-identified as White, and 15.83% (n=19) as Black. As for the reason why they decided not to get the vaccine, 48.76% (n=59) stated that the reason was because the vaccine would make them sick. 14.05% (n=17) did not trust the vaccine and 8.25% (n=10) thought the vaccine is being used to manipulate people (see Table 1.) An overwhelming 61.34% (n=73) also said that nothing would change their mind about getting vaccinated. 84% (n=104) of the respondents stated they would not be interested in getting more information about COVID-19 vaccine, while 66.67% (n=82) said they will not consider getting vaccinated in the future (see Table 2.).

**Conclusion:**

Vaccine hesitancy affects public health and the well-being of communities. Importantly, vaccine hesitancy is a decision made by an individual affected by a myriad of factors permeating their environment. Additionally, a person is more likely to get vaccinated if they perceive vulnerability, and ultimately, if they trust that a scientific advancement.

**Disclosures:**

**All Authors**: No reported disclosures

